# The Potential Exploitation of the Mediterranean Invasive Alga *Caulerpa cylindracea*: Can the Invasion Be Transformed into a Gain?

**DOI:** 10.3390/md14110210

**Published:** 2016-11-15

**Authors:** Loredana Stabili, Simonetta Fraschetti, Maria Immacolata Acquaviva, Rosa Anna Cavallo, Sandra Angelica De Pascali, Francesco Paolo Fanizzi, Carmela Gerardi, Marcella Narracci, Lucia Rizzo

**Affiliations:** 1 Dipartimento di Scienze e Tecnologie Biologiche ed Ambientali, Università del Salento, Via Prov.le Lecce Monteroni, 73100 Lecce, Italy; simona.fraschetti@unisalento.it (S.F.); sandra.depascali@unisalento.it (S.A.D.P.); fp.fanizzi@unisalento.it (F.P.F.); lucia.rizzo@unisalento.it (L.R.); 2 Istituto per l’Ambiente Marino Costiero, Unità Operativa di Supporto di Taranto, CNR, Via Roma 3, 74123 Taranto, Italy; maria.acquaviva@iamc.cnr.it (M.I.A.); rosanna.cavallo@iamc.cnr.it (R.A.C.); marcella.narracci@iamc.cnr.it (M.N.); 3 CoNISMa—Consorzio Nazionale Interuniversitario per le Scienze del Mare, Piazzale Flaminio 9, 00196 Rome, Italy; 4 Istituto di Scienze delle Produzioni Alimentari, Unità Operativa di Supporto di Lecce, via Prov.le Lecce-Monteroni, 73100 Lecce, Italy; carmela.gerardi@ispa.cnr.it

**Keywords:** seaweed, *C. cylindracea*, NMR spectroscopy, lipidic extract, antioxidant activity, antimicrobial activity, fatty acids

## Abstract

Recently, there is a growing interest towards the development of strategies for invasive seaweed control and exploitation as source of secondary metabolites. Here, we investigated the potential of exploitation in biotechnology and recycling options in eradication programs of the lipidic extract of the Mediterranean invasive seaweed *Caulerpa cylindracea* (Chlorophyta). The chemical characterization was carried out by means of multinuclear and multidimensional NMR spectroscopy. The fatty acid profile of *C. cylindracea* assessed the presence of several types of molecules known for antioxidant activity such as carotenoids, chlorophylls, pheophytins, and sterols. The NMR spectroscopy showed also the characteristic signals of saturated, unsaturated, and free fatty acids as well as other metabolites including the biopolymer polyhydroxybutyrate. The lipidic extract exerted an antioxidant activity corresponding to 552.14 ± 69.13 mmol Trolox equivalent/g (ORAC) and to 70.3 ± 2.67 mmol Trolox equivalent/g (TEAC). The extract showed an antibacterial activity against several *Vibrio* species, suggesting its potential use in the control of diseases in mariculture. Our results show that *C. cylindracea*, representing a critical hazard in coastal areas, could be transformed into a gain supporting specific management actions to reduce the effects of human pressures.

## 1. Introduction

Marine organisms are rich sources of structurally novel and biologically active metabolites, exhibiting numerous interesting biological effects and thus constituting valuable opportunities for drug discovery. Recently, several studies on the growing problem of non-indigenous species have proven that the knowledge of marine bioactive compounds may indicate the capacity of non-indigenous species to invade new systems [1,2,3,4,5,6,7,8,9]. In a recent review, Mollo et al. [10] showed that the commercial use of the natural products obtainable from marine bioinvaders might also be considered as an effective option for reducing their impact on marine ecosystems.

In coastal habitats, macroalgae are abundant and represent a source of bioactive metabolites exhibiting numerous promising and remarkable biological activities capable of influencing the abundance, distribution, and survival of marine organisms [11,12]. Seaweeds are known for their richness in polysaccharides, minerals, and certain vitamins [13]. Marine algae have been also recognized as potential source of antioxidants [14,15] and traditionally consumed as a readily available whole food, especially among coastal communities [16,17,18]. Fatty acids and enzymatic and non-enzymatic antioxidant properties of have been investigated in *Caulerpa* species [19,20,21,22]. Macroalgae also contain bioactive substances like polysaccharides, proteins, lipids, and polyphenols, with antibacterial, antiviral, and antifungal properties [23].

Seaweeds have been prescribed for several diseases in different Asian traditional medical systems [18]. In recent years, pathogenic bacteria resistant to multiple drugs have become a worldwide emergency. The discovery of new antibacterial compounds, as suitable substitutes to conventional antibiotics, might be a possible solution to this problem. Seaweeds could represent a potential source of new antimicrobial compounds [24]. Ballesteros and Uriz [25] have screened several marine macrophytes from the Central Mediterranean to evaluate the production of antibacterial, antifungal, compounds, founding, among the Chlorophyta, with the maximum level of activity found in the order Bryopsidales. In particular, species belonging to the genus *Caulerpa* show interesting antibacterial activity compared to other groups of algae [26]. A study conducted on the aqueous extract of *Caulerpa racemosa* collected in India (Gulf of Mannar) showed a pronounced antibacterial activity against different pathogenic organisms [27]. Moreover, the methanol extract of *C. racemosa* was found to exhibit significant activity against the test pathogens *Staphylococcus aureus*, *Escherichia coli*, and *Enterobacter aerogenes* [28].

In the present study, we focused on the Mediterranean non indigenous seaweed *Caulerpa cilindracea* (Sonder) [29], previously known as *C. racemosa* var. *cylindracea* (Sonder) Verlaque, Huisman and Boudouresque [30]. The vector of introduction in the Mediterranean Sea is unknown. It was initially hypothesized to be a Lessepssian immigrant, or a hybrid between *C. racemosa* var. *turbinata* and an unknown tropical variety [31] until molecular analyses identified a possible source population around the Australian-Pacific area [30,31,32]. It can invade different habitats, grow rapidly, and spread though fragmentation and propagation [33,34], leading to profound structural and functional alterations of native benthic assemblages and fish metabolism [9,35,36]. In the Mediterranean, the production of secondary metabolites by the alga changes seasonally and the highest levels have been observed during the period of vegetative algal growth (summer and autumn) [37]. Moreover Blažina et al. [20] observed that in *C. racemosa* from sheltered sites generally small variations in total lipids were found.

Here the chemical characterization of *C. cylindracea* lipidic extract was carried out by means of the advanced analytical technique of multinuclear and multidimensional NMR spectroscopy. In addition, the presence of secondary metabolites in the seaweed extract including bioactive compounds with antimicrobial and antioxidant activities was assessed. Since eradication has been recently suggested as a powerful action to protect areas highly impacted by invasive species, the secondary metabolites were investigated with the perspective of using *C. cylindracea* in eradication programs to support biotechnology and recycling options.

## 2. Results

### 2.1. NMR Spectoscopy

**Figure 1:**
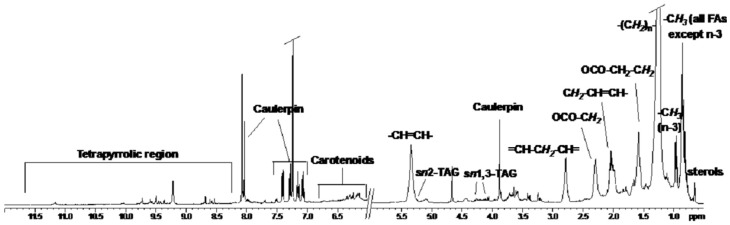
^1^H NMR in CDCl_3_ of *C. cylindracea* lipidic extract.

The 1D ^1^H (Figure 1) spectrum in spectrum in CDCl_3_ of the algal lipid fractions shows the characteristic signals of fatty acids (FAs), such as saturated (SAFAs) and unsaturated (UFAs) fatty acids, as well as caulerpin and other metabolites. At low frequencies the singlet at 0.66 ppm, which in the ^1^H–^13^C HSQC shows cross peak correlation with the carbon at 11.8 ppm, was attributed to a methyl group of sterols. 

The signals at 0.85 ppm were assigned to the methyl groups of all FAs except ω3, which give a triplet at 0.95 ppm. The large signal at 1.21 and 1.50 ppm was attributed to alkyl chain and methylene group in β position to C=O of all fatty acids. The large signals between 1.94 and 2.12 ppm and the signals at 5.35 ppm were attributed to methylene groups in α position with respect to vinyl groups and vinyl groups of all UFAs, respectively. The methylene groups in α to C=O of all FAs resonate at 2.29 ppm. The bisallylic protons, characteristic of fatty acids with two or more double bonds, give signals at 2.78 ppm.

In addition, the characteristic spin system of poly-β-hydroxybutyrate (PHB) was identified in extract by 2D ^1^H COSY spectra (two signals at 2.45 and 2.58 ppm, attributed to the methylene group, coupled with the methyl group at 1.26 ppm and the methine at 5.23 ppm). By 1D ^1^H and 2D ^1^H COSY spectra of all fractions different pattern signals for esterified glycerols were observed. The coupling system connecting the multiplet at 5.25 ppm and the doublet of doublet at 4.27 and 4.12 ppm was assigned to the C*H sn2* and the two C*H*_2_ *sn1* and *sn3* of triacylglycerol. However, by 1D ^13^C-NMR a higher intensity of signals at 179 ppm, attributed to carbonyl groups (C=O) of free fatty acids (FFAs) with respect to the signals in the range 173–174 ppm assigned to carbonyl groups of esterified fatty acids (EFAs) was observed.

In the 1D ^1^H NMR spectrum at high frequencies the aromatic signals of caulerpin were observed. The doublets at 7.41 and 7.28 ppm and the multiplets at 7.16 and 7.07 ppm were assigned to the bis-indolic ring of caulerpin, whereas the singlets at 9.22, 8.04, and 3.88 ppm were assigned to NH of bis-indole, =CH- of central ring and ester methyl groups, respectively. The signals in the high frequencies region between 11.2 and 8.5 ppm were attributed to methine bridge protons of chlorophyll and pheophytins. These signals were confirmed by the presence at negative value of chemical shift (−1.5 to −2.00 ppm) of peaks corresponding to the N-H protons of the tetrapyrrole ring. The signals in the range 6.00 to 6.70 ppm were assigned to conjugated double bonds of carotenoids. Isomers that are all *trans* are prevalent in fresh matter and the quantities of *cis* isomers increase after thermal processing.

### 2.2. Antioxidant Activity

The antioxidant activity of the lipidic extract from *C. cylindracea* assayed by TEAC and ORAC assays is reported in Table 1. Antioxidant capacity of the seaweed extract measured by the ORAC assay was eight times higher than the activity measured by the TEAC assay.

### 2.3. Antimicrobial Activity

The results of antimicrobial activity of *Caulerpa cylindracea* lipidic extract towards the utilized microbial strains are shown in Table 2. Yeasts were not affected in their growth by *C. cylindracea* lipidic extract. Moreover, the extract did not show antibacterial activity against *Enterococcus* sp., *Escherichia coli*, *Staphylococcus* sp., and *Streptococcus* sp. By contrast, the degree of inhibition produced by the lipidic extract on some *Vibrio* species was quantified. In particular, using discs with 100 μL of algal extract, *V. fischeri*, *V. inusitatus*, and *V. litoralis* were the most inhibited (diameter of growth inhibition = 0.9 cm). A lower percentage of inhibition was measured on *V. aestuarinus* (0.85 cm), *V. mediterranei* (0.8 cm), and *V. vulnificus* (0.8 cm).

## 3. Discussion

In the present study, the lipidic extract of the Mediterranean invasive alga *C. cylindracea* was analyzed by multinuclear and multidimensional NMR spectroscopy and its antioxidant, antibacterial, and antifungal activities have been evaluated.

Some important issues can be inferred from the obtained results:

By the NMR analysis the ^1^H NMR spectrum in CDCl_3_ of *C. cylindracea* algal lipid fraction interestingly, for the first time, showed the presence of polyhydroxybutyrate (PHB), a natural, linear biodegradable, and biocompatible polymer belonging to the polyesters group of bioplastics. PHB is synthesized by microorganisms as a form of energy-storage granules and utilized when the other energy sources are not available [38]. PHB has been already evidenced in the red algae *Plocamium cartilagineum* (Linnaeus) Dixon [39], *Gracilariopsis longissima* [17], and *Cladophora rupestris* [40]. Due to the rapid degradation and the non-toxicity of the final products, PHB represents an important ingredient for the production of polymers used in various biotechnological applications such as in the production of several medical devices and compostable plastic [41,42]. As crude oil reserves decrease throughout the world, petroleum based plastics are becoming less economical. Moreover, petroleum based plastics cannot be considered environmentally friendly due to their resistance to natural or biological decomposition. In this framework, *Caulerpa cylindracea* could represent one of the alternatives for the production of bioplastics because it is an excellent renewable resource due to its high bioinvasion potential and high growth rate. The technology development for seaweeds-based bioplastics are still in the research phase. The challenge is to have significant advancements in the bioplastics industries to make seaweed bioplastics a concrete alternative in the future as already underlined in a recent review by Noreen et al. [43].

The NMR analysis besides other metabolites also showed the characteristic signals of SAFAs, UFAs, FFAs, and EFAs. NMR has indeed the desirable property of providing a global profiling tool for monitoring rapidly the molecular components of marine organisms [44]. Fatty acids are compounds usually bound to other molecules including glycerol, sugars, or phosphate groups thus constituting the lipids. As already reported for other algal lipid extracts [39,45], the presence of monogalactosyl diacylglycerol (MGDG) was recognized in *C. cylindracea* by NMR spectroscopy. Our results are in accordance with other studies on *C racemosa* and other seaweeds confirming their nutritional value [21]. In particular, seaweeds are low in calories, have high content of dietary fiber, are a good source of polyunsaturated fatty acids DHA and EPA, and may contain proteins with an amino acid profile of interest [46]. Apart from the importance of fatty acids for diet, their ability to interfere with bacterial growth and survival has been established in several organisms including seaweeds [40,47]. This is noteworthy since in this study we observed for the first time an antibacterial activity of *C. cylindracea* against *Vibrio aestuarianus*, *V. fischeri*, *V. inusitatus*, *V. litoralis*, *V. mediterranei*, and *V. vulnificus*. This is a critical result considering that aquaculture is emerging as the fastest growing food-producing industry in the world because of the increasing demand for food fish consumption. However, the intensive culture of food fish has led to outbreaks of various microbial diseases, resulting in annual economic losses to the aquaculture industry estimated at billions of dollars worldwide [48]. Bacteria, mainly from the genus *Vibrio*, have been identified as the etiological agents responsible for the most common disease outbreaks in fish and shellfish causing mortality in several countries [49]. Disease outbreaks caused by several *Vibrio* species including *V. aestuarianus*, *V. mediterranei*, and *V. vulnificus* are called vibriosis [50,51,52]. Treatments of infected fish with antibiotic- medicated food are a common practice but have led to the development of bacterial antibiotic resistance, resulting in a higher dose requirement for effective control and a consequent increase of chemical residues released into the environment posing serious risks to animal and human health. Therefore, there is currently an increasing request for more environment-friendly alternatives to conventional antibiotics with similar or enhanced properties for aquatic animals. In the last 20 years, there has been an increasing interest in using various seaweed extracts as prophylactic and/or therapeutic agents in aquaculture [53]. Thus, the ability of the *C. cylindracea* lipidic extract to act against aquatic pathogens at a concentration of 5 mg/mL, evidenced in the present study, highlights its potential exploitation as a source of antibacterial compounds, of great importance in the control of disease in the mariculture industry, which is a largely unexplored field of research.

The seaweed extract did not show activity against the yeasts *C. albicans* and *C. glabrata*, and against the bacterial strains *Enterococcus* sp., *E. coli*, *Staphilococcus* sp., *Streptococcus* sp. It is well known that the antibacterial activity can be affected by many factors and that the method of extraction is one of the most important ones since different compounds, capable of acting on different bacterial strains, can be obtained by different extraction procedures [53,54]. The substances isolated from green, brown, and red algae showing powerful antimicrobial activity belong to different classes and include polysaccharides, fatty acids, phlorotannins, pigments, lectins, alkaloids, terpenoids, and halogenated compound. As an explanation, *C. racemosa* ethyl acetate extract exhibits antibacterial activity against *Enterococcus faecalis*, *Staphylococcus aureus*, *Bacillus cereus*, and *Escherichia coli* [54,55]. By contrast, methanolic extracts of *C. racemosa* shows antibacterial activity against *Klebsiella pnemoniae*, *Enterobacter aerogens*, *Pseudomnas aeruginosa*, *Micrococcus luteus*, *Enterobacter faecalis*, *Streptococcus faecalis*, *Staphylococcus aureus*, and *Bacillus subtilis* [26,38]. In the present study, we evaluated the antibacterial activity of the *C. cylindracea* lipidic extract but further studies are needed to evaluate whether the crude extract or aqueous extract lead to evidence other relevant biological activities.

The seaweed lipidic extract exerts an important antioxidant activity. Even though a comparison of antioxidant activity across seaweeds is difficult due to seasonal, environmental, and genetic variations, the obtained ORAC mean value of 552 μm Trolox equivalent/g extract for *C. cylindracea* is similar to those of lipid-soluble extracts of *Macrocystis pyrifera* (462 μm Trolox equivalent/g extract) and *Ecklonia radiata* (363 μm Trolox equivalent/g extract). This is noteworthy since these seaweeds, with powerful activity, have been recently proposed as potential sources of natural antioxidants instead of chemical antioxidant [56].

The TEAC and ORAC methods were simultaneously utilized in the present work since many studies stress the need of adopting at least two approaches to take into account the different mechanisms of antioxidant action [57,58], as single assay may disregard some radical sources or all antioxidants [59]. The methods utilized here are among the most popular assays and differ for their reaction mechanisms: TEAC is an electron transfer (ET) based method, while ORAC is based on hydrogen atom transfer (HAT) method. Other studies have already been conducted on the antioxidant activity of *C. racemosa* [21,60]. The utilized methodology is generally different across studies so that a comparison of seaweed antioxidant activity is challenging. The results obtained in the present study indicate that the ORAC values were higher in the measurement of antioxidant capacity of the lipidic extract of *C. cylindracea* than the TEAC. These results are in accordance with literature data indicating that carotenoids contain a chain of isoprene residues bearing numerous conjugated double bonds and are mostly involved in the scavenging of two of the reactive oxygen species, singlet molecular oxygen (O_2_), and peroxyl radicals (ROO•). The HAT-based antioxidant capacity assays, like ORAC, utilize a radical initiator to generate peroxyl radicals and measure the antioxidant activities of antioxidant molecules against peroxyl radicals [61]. Then the antioxidant activity of carotenoids is higher when measured with ORAC assay on comparison to TEAC assay. By contrast, the antioxidant activity of phenolic acids is similar using both methods [62].

Interestingly, the characterization of *C. cylindracea* lipidic extract by 1D and 2D NMR spectroscopy assessed the presence of several types of molecules known for their antioxidant activity. Carotenoids, which produce signals in the 6.00–6.70 ppm range, were identified as well as the signals of chlorophylls and its thermal by-product, metal-free pheophytins. Both chlorophylls and pheophytins have demonstrated protective activity against auto-oxidation of vegetable oils in the dark [63,64]. Endo et al. [63] suggested that chlorophyll derivatives may act as electron donors as evidenced by their ability to reduce free radicals such as 1,1,diphenyl-2-picrylhydrazyl. Moreover, chlorophyll *a* was shown to act synergistically with vitamin E by quenching tocopherol radicals, thereby enhancing the observed antioxidant effects of vitamin E [65,66]. The ORAC specificity, the medium polarity of the extraction solvent, together with the content of carotenoids, chlorophylls, pheophytins, and sterols could explain the higher ORAC value of the *Caulerpa* lipidic extract compared to those obtained by TEAC according with other studies on *Caulerpa* [67,68]. Previous studies highlighted greater antioxidant activities of the green seaweed *C. racemosa* from Malaysian North Borneo compared to other brown and red seaweeds, observing that the phenolic compounds were involved in the antioxidant activity [69]. Further studies conducted on *C. racemosa* highlighted that the major contributors to the antioxidant activities are medium polarity phenolic compounds [70,71]. The further identification, characterization, and isolation of the compounds involved in *C. cylindracea* antioxidant activity might contribute to the employment of algal extracts in disease treatments related to oxidative stress, taking into account that, recently, an interest in natural antioxidants has increased because they are safer than synthetic antioxidants.

In conclusion, our findings open a new research area on the possible employment of the examined seaweed in the biotechnological field as source of bioactive natural products including antibiotics, antioxidants, fatty acids, and PHB potentially carrying benefits to human and marine organisms’ health. At present, eradication of recent alien introduction [72] has been proposed as a promising management action to assist the recovery of highly invaded areas under protection regime such as Marine Protected Areas. In this framework, the overproduced biomass of the invasive seaweed *C. cylindracea* could transform into a gain offering a potential tool with recycling options in eradication programs.

## 4. Materials and Methods 

### 4.1. Study Site and Species Collection

*Caulerpa cylindracea* was collected in the Marine Protected Area of Torre Guaceto located in the Mediterranean Sea (Southern Adriatic Sea, Brindisi, Italy) at the depth of 8–10 m on the rocky bottom, during the season of maximum growth of the species when the alga dominates the benthic assemblages forming continuous dense meadows across the areas. *Caulerpa cylindracea* shows a seasonal cycle with a period of vegetative growth approximately between June and November alternated with a period of vegetative rest (a quasi-complete withdrawal) approximately from December to May [73,74]. Seasonal variations in the growth rate were highly significant: during the maximum development the biomass is 82 ± 3 g·DW·m^−2^ and length of stolons 1162 ± 86 m·m^−2^. By contrast, during the minimum development the biomass is reduced to 0.3 ± 0.1 g·DW·m^−2^ and length of stolons to 3 ± 1 m·m^−2^ [74]. Three replicates of about 500 g of fresh material were collected by SCUBA diving. All the harvested material was transferred into aseptic containers to the laboratory under controlled temperature (4 °C). The species was identified on the basis of morphological features. In detail, the following morphological features of the thalli were analyzed: height, width and attachment to stolons of the fronds; height, diameter, shape, and arrangement of the ramuli; diameter of the stolons; length, width, spacing, and morphology of the rhizoids. Algal morphological features fit the description given by several authors for the invasive species *C. cylindracea* [29,36,75,76,77].

### 4.2. Preparation of Lipidic Extracts from the Macroalga

Algae samples were cleaned of epiphytes and other marine organisms with a mixture of ethanol and (40%) and sodium hypochlorite (1%) for 10 s [78] and necrotic parts were removed. The samples were further rinsed with sterile water to remove any associated debris. The freshly cleaned material was air-dried and powdered, then 3 g of each sample was extracted in 150 mL of chloroform/methanol (2:1 at 55–60 °C for 24 h) using a soxhlet apparatus. Extraction solvents were evaporated under vacuum at controlled temperature. 5 mg of extract was then dissolved in 1 mL of absolute ethanol (95%; by J.T. Baker), and assayed for antimicrobial and antioxidant activity. 

### 4.3. NMR Spectroscopy

The lipid fraction of *C. cylindracea* was characterized by ^1^H and ^13^C 1D and 2D NMR spectroscopy with the same methodology already reported in Stabili et al. [47]. 1D ^1^H and ^13^C, 2D ^1^H *J*res, ^1^H COSY, ^1^H–^13^C HSQC, and ^1^H–^13^C HMBC spectra were recorded at 298.15 K on a Bruker Avance III 400 MHz spectrometer using CDCl_3_ as solvent and chemical shift was referenced to TMS by the residual protic solvent peaks as internal references ((^1^H = 7.24 ppm; ^13^C = 77.0 ppm). High resolution ^13^C-NMR spectra were acquired semi-quantitatively [79], with short relaxation times, and high number scans, to achieve sufficient S/N ratio to calculate the integrals. The following parameters were used: 64 K data points, spectral width of 20,161.291 Hz, 16 K scans with a 0.5 s repetition delay, and 60° at ^13^C excitation pulse. The acquisition and processing of spectra were performed using the software Topspin 2.1 (Bruker Biospin). Resonances of fatty acids and metabolites were assigned on the basis of literature data [40,47,80,81].

### 4.4. Antioxidant Activity

#### 4.4.1. Oxygen Radical Absorbance Capacity Assay (ORAC)

For ORAC the method of Davalos et al. [82] was used. Extracts were diluted with 75 mM phosphate buffer (pH 7.4). The assay was carried out in black-walled 96-well plates (Greiner-Bio One) and each well contained a final volume of 200 μL. To each well 20 μL of extract and 120 μL of fluorescein (FL; 70 nM final concentration) were added and the plate was incubated at 37 °C for 15 min. The AAPH (60 μL; 12 mM final concentration) was added to each well and fluorescence intensity was estimated using an Infinite200 Pro plate reader (Tecan, Männedorf, Switzerland), every minute for a total of 80 min using an excitation wavelength of 485/9 nm and an emission wavelength of 535/20 nm. A standard curve was constructed using 6-hydroxy-2,5,7,8-tetramethylchroman-2-carboxylic acid (Trolox, Sigma-Aldrich, Oakville, ON, Canada, 1.5–10.5 μM). A blank (fluorescein + AAPH) using phosphate buffer instead of the antioxidant solution was carried out in each assay. Results were determined by using Magellan v 7.2 software (Tecan, Männedorf, Switzerland), on the basis of the difference in area under the curve between the control and the sample and expressed as μmoles of Trolox equivalents (TE) per g of lipidic extract. All the reaction mixtures were prepared in triplicate and at least three independent assays were performed for each sample.

#### 4.4.2. Trolox Equivalent Antioxidant Capacity Assay (TEAC)

The TEAC assay was performed as previously described by Re et al. [83] with minor modifications to adapt the assay to a microplate reader. Briefly 2,2′-azinobis (3-ethylbenzothiazoline-6-sulfonic acid) diammonium salt (ABTS, Sigma-Aldrich) radical cations were prepared by mixing an aqueous solution of potassium persulfate 2.45 mM (final concentration) and an aqueous solution of ABTS 7 mM (final concentration) and allowing it to stand in the dark at room temperature for 12–16 h, before use. The ABTS radical cation solution was diluted in PBS (pH 7.4) to an absorbance of 0.40 at 734 nm ± 0.02; this value was adopted to obtain about 80% of maximum inhibition of the blank absorbance using the highest concentration of the Trolox standard curve. Trolox was used as antioxidant standard and a standard calibration curve was constructed for Trolox (0–16 μM). After addition of 200 μL of diluted ABTS to 10 μL of Trolox standard or extracts diluted in PBS, in each well of a 96 well-plate (Costar), the absorbance reading at 734 nm was taken 6 min after initial mixing using an Infinite200 Pro plate reader (Tecan, Männedorf, Swizerland). Appropriate solvent blanks were run in each plate. The lipidic extract was assayed in at least three separate dilutions and in triplicate. The percentage inhibition of absorbance at 734 nm is calculated and plotted as a function of concentration of Trolox and the TEAC value expressed as Trolox equivalent (in μmolar) per g of lipidic extract, using Magellan v 7.2 software.

### 4.5. Test Microorganisms

The antibacterial activity was tested on six human pathogenic microbial strains: *Candida albicans*, *Candida glabrata*, *Enterococcus* sp., *Escherichia coli*, *Staphilococcus* sp., *Streptococcus* sp., and several *Vibrio* strains isolated from marine environment: *Vibrio aestuarinus*, *Vibrio campbellii*, *Vibrio carchariae*, *Vibrio diazotrophicus*, *Vibrio fischeri*, *Vibrio fluvialis*, *Vibrio furnissii*, *Vibrio harveyi*, *Vibrio inusitatus*, *Vibrio litoralis*, *Vibrio mediterranei*, *Vibrio natriegens*, *Vibrio ordalii*, *Vibrio salmonicida*, *Vibrio splendidus II*, *Vibrio vulnificus*. The tested strains were isolated and identified from seawater samples of the Mar Piccolo of Taranto as previously reported by Cavallo et al. [84] and Stabili et al. [85].

### 4.6. Antimicrobial Activity

Antimicrobial activity was evaluated using the Kirby Bauer method [86]. Sterile paper discs, 6 mm in diameter (AA; Whatman International Ltd., Maidstone, Kent, UK), were impregnated with 10, 20, 30, 40, 60, 80, 100 μL of each extract and left to air-dry at room temperature for 4 h, as already described by Cavallo et al. [87]. By contrast, discs impregnated with an equivalent volume of carrier solvent were used as controls. Moreover, an ‘extraction blank’ as a negative control (MeOH/CHCl_3_ extraction with no algae, dry, then suspended in ethanol) was also used. For each assay, sterile medium-agar plates opportune for each selected bacterial and fungal strain tested were seeded with 100 μL of microbial suspension (about 10^8^ CFU mL^−1^) [88,89], using a sterile swab to give a uniform covering. Impregnated discs and controls were laid onto the agar surface, the *Vibrio* plates were then incubated for 24 h at 30 °C, the other tests were conducted at 37 °C. The clear zone around the discs was evidence of antibacterial activity. The diameter of the microbial growth inhibition was taken as the diameter of the clear zone (measured in centimeters).

## Figures and Tables

**Table 1:** Antioxidant activity of *C. cylindracea* lipidic extract assayed by TEAC and ORAC assays.

ORAC value	552.14 ± 69.13 μmol Trolox Equivalent/g extract
TEAC value	70.03 ± 2.67 μmol Trolox Equivalent/g extract

Data are the mean ± SD (*n* = 3).

**Table 2:** Antimicrobial activity of *C. cylindracea* lipidic extract.

Microbial Strain	Diameter of Growth Inhibition (cm)						
	10 μL	20 μL	30 μL	40 μL	60 μL	80 μL	100 μL
*Candida albicans*	0	0	0	0	0	0	0
*Candida glabrata*	0	0	0	0	0	0	0
*Enterococcus* sp.	0	0	0	0	0	0	0
*Escherichia coli*	0	0	0	0	0	0	0
*Staphilococcus* sp.	0	0	0	0	0	0	0
*Streptococcus* sp.	0	0	0	0	0	0	0
*Vibrio aestuarianus*	0.7	0.7	0.7	0.7	0.7	0.8	0.85
*Vibrio campbelli*	0	0	0	0	0	0	0
*Vibrio carchariae*	0	0	0	0	0	0	0
*Vibrio diazotrophicus*	0	0	0	0	0	0	0
*Vibrio fischeri*	0.8	0.8	0.8	0.8	0.8	0.8	0.9
*Vibrio fluvialis*	0	0	0	0	0	0	0
*Vibrio furnissi*	0	0	0	0	0	0	0
*Vibrio harveyi*	0	0	0	0	0	0	0
*Vibrio inusitatus*	0.8	0.8	0.8	0.8	0.8	0.9	0.9
*Vibrio litoralis*	0.8	0.8	0.8	0.8	0.8	0.8	0.9
*Vibrio mediterranei*	0	0	0.7	0.7	0.7	0.8	0.8
*Vibrio natriegens*	0	0	0	0	0	0	0
*Vibrio ordalii*	0	0	0	0	0	0	0
*Vibrio salmonicida*	0	0	0	0	0	0	0
*Vibrio splendidus II*	0	0	0	0	0	0	0
*Vibrio vulnificus*	0.8	0.8	0.8	0.8	0.8	0.8	0.8
